# Self-medication among medical students at the Copperbelt University, Zambia: A cross-sectional study

**DOI:** 10.1016/j.jsps.2021.10.005

**Published:** 2021-10-25

**Authors:** Owen Banda, Pipina Anna Vlahakis, Victor Daka, Scott Kaba Matafwali

**Affiliations:** a School of Medicine, Public Health Department, Copperbelt University, Ndola, Zambia; b School of Medicine, Basic Science Department, Copperbelt University, Ndola, Zambia

**Keywords:** Self-medication, Rational drug use, Zambia, Medical students

## Abstract

**Background:**

Self-medication is a common practice and cause for concern globally. There is a paucity of information regarding students’ self‑medication in Zambia. Therefore, this study aimed at determining the self‑medication practices among the medical students at Copperbelt University.

**Methods:**

A descriptive cross-sectional study was conducted among 334 students. Data was collected using a semi-structured questionnaire, entered in excel, cleaned, and exported to SPSS version 21 for statistical analysis. Univariate analysis using Chi-Square or Fishers Exact test was performed. Independent predictors of self-medication practices were determined using logistic regression. Adjusted Odds ratios (AOR) and their 95% confidence intervals are reported.

**Results:**

A total of 334 medical students, with slightly more females (50.3%, n = 168), participated in the study. Of these 61.1% (n = 204) reported self-medicating. Reasons for self-medicating were the presence of long queues at health facilities and lack of time to visit the hospital. Only the year of study was independently associated with self-medicating with those in their 4th year of study being more likely to self-medicate [AOR:3.43, 95% CI: 1.52–7.73].

**Conclusion:**

Students should be educated on the consequences of self-medication practices especially the rational use of antibiotics.

## 1 Introduction

Self-medication is the use of medicines to treat self-diagnosed conditions or symptoms without the advice and prescription of a doctor (Hughes et al., 2001, World Health Organization. Guidelines for the Regulatory Assessment of Medicinal Products for use in Self-Medication, 2000). This human behaviour involves the self-recognition of symptoms in which the individual then uses medicine to self-administer treatment. Self-medication has been said to reduce the cost of health care and allows health workers to concentrate on emergency cases, but the harm and side effects of self-medication can be so severe that they may in turn become emergencies (Hughes et al., 2001) (see Tables 1 and 2).

**Table 1:** Prevalence of self-medication by demographic characteristics of participants.

**Variable**	**Overall**	**Self-medication**		
	n = 334	Yes (n = 204)	No (n = 130)	p value
				
**Sex**				
Male	166 (49.7%)	105 (51.5%)	61 (46.9%)	0.418
Female	168 (50.3)	99 (48.5%)	69 ((53.1%)	
				
**Age Groups**				
18–24 years	200 (59.9%)	122 (59.8%)	78	0.972
>24 years	134 (40.1%)	82 (40.2%)	52	
				
**Marital Status**				
Single	315 (94.3%)	192 (94.1%)	123 (94.3%)	0.519
Married	17 (5.1%)	10 (4.9%)	7 (5.4%)	
Divorced	2 (0.6%)	2 (1.0%)	0 (0.0%)	
				
**Year of Study**				
2nd	59 (17.7%)	33 (16.2%)	26 (20.0%)	0.038
3rd	55 (16.5%)	43 (21.1%)	12 (9.2%)	
4th	43 (12.9%)	29 (14.2%)	14 (10.8%)	
5th	53 (15.9%)	29 (14.2%)	24 (18.5%)	
6th	124 (37.1%)	70 (34.3%)	54 (41.5%)	
				
**Religion**				
Christian	330 (98.8%)	202 (99.0%)	128 (98.5%)	0.644
Muslim	4 (1.2%)	2 (1.0%)	2 (1.5%)

**Table 2:** Responses of students for self-medicating.

**Variable**	**Yes**	**No**
	n (%)	n (%)
Long queues at the hospital	251(75.1)	83(24.9)
Nurse and Doctors are rude to medical students	76(22.8)	258(77.2)
Medical Students have enough knowledge about health problems and can treat themselves	107(32.0%)	227(68.0)
There is a shortage of medicines in hospitals	113(33.8)	221(66.2)
There is a long distance to the hospital	122(36.5)	212(63.5)
Lack of time to visit the hospital	218(65.3)	116(34.7)

Several studies have assessed the problem of self-medication globally and found that the prevalence is high in many regions (Ayalew, 2017, Gogazeh, 2020, Yousef et al., 2007). The Covid-19 pandemic has also amplified this problem with several studies showing that many people self-medicate (Quispe-Cañari et al., 2021, Sadio et al., 2021). Several studies have found that antibiotics and over-the-counter (OTC) medicines such as pain killers are the most used for self-medication and easily accessed (Horumpende et al., 2018, Pirzadeh and Mostafavi, 2014, Van et al., 2020). Antibiotic use is particularly important because it is one of the drivers of the development of antimicrobial resistance (AMR) and it is currently estimated that AMR complications will lead to about 10 million deaths by 2050 (World Health Organization, 2015). The low-income and middle-income countries (LMICs) are more affected by AMR mainly due to insufficient infection prevention control measures and the increased burden of infectious diseases (Mueller and Östergren, 2016).

The practice of self-medication has been studied among students in several countries. A study by Esan (Esan et al., 2018) found that about 71% of the students self-medicated with antibiotics and analgesics. Studies by Zeru (Zeru et al., 2020) in Ethiopia and Hashemzaei (Hashemzaei et al., 2021) in Iran found a similar self-medication prevalence rate of about 50% among university students. In these studies, fever and headache were the most reported common complaint related to self-medication. Prior experience with the drugs was the most common reason for self-medicating in both studies. Several other studies in different populations have revealed factors such as availability of drugs, the level of education, family, and the exposure to advertisements as predictors of self-medication (Araia et al., 2019, Ayalew, 2017, Yousef et al., 2007).

Few studies have explored self-medication in Zambia. For example, a study by Kalungia et al (2016) found that non-prescription sale and dispensing of antibiotics was widespread in Zambia with self-medication as one of the drivers. There is a paucity of information regarding self‑medication in Zambia among students. Therefore, we carried out a study aimed at determining the self‑medication practices among the medical students at Copperbelt University.

## 2 Material and methods

### 2.1 Study site

The study was conducted at the Copperbelt University, Michael Chilufya Sata School of Medicine. The School is the largest school of medicine in the Northern region of Zambia and is the second largest in Zambia. It has a student population of approximately 2500 students mostly undergraduates and a much smaller population of postgraduate students.

### 2.2 Study population and study design

A descriptive cross-sectional study was conducted among undergraduate students at the Copperbelt University Michael Chilufya Sata School of Medicine. Students were consecutively enrolled after providing informed consent to participate in the study. To ensure all the students had an equal chance of participation, the study was introduced to all the undergraduate students from 2nd Year to 6th Year.

### 2.3 Sample size calculation

To our knowledge, this is the first study investigating self-medication practices among undergraduate students in Zambia. Employing methods for determining the sample size of cross-sectional studies as described by Pourhoseingholi and others (Pourhoseingholi et al., 2013), we assumed a conservative prevalence of the practice of self-medication estimate of 50%, 95% confidence interval, the precision of 5%, and extrapolated to a student population of 2500. We determined the minimum required sample size as 333. A total of 334 participants were subsequently enrolled.

### 2.4 Data collection

Data were collected using a semi-structured questionnaire comprising four sections namely, section A on demographics, section B on knowledge regarding self-medication (8 questions), section C on practice towards self-medication (6 questions), and section D on factors associated with self-medication (7 questions). Content validation was done by distributing the questionnaire among faculty at the Copperbelt University School of Medicine. Face validation was conducted through a pilot study among students at the Ndola School of Nursing. The data collected from the pilot study were censored from the final analysis. Feedback from both face and content validation exercises was used to optimise the questionnaire to ensure logic and consistency were maintained.

### 2.5 Data analysis

Data were entered and cleaned in Microsoft Excel (Microsoft Corp, Redmond, WA, USA) and exported to SPSS version 21 (IBM SPSS Inc., Chicago, IL, USA) for statistical analysis. Demographic variables were presented as descriptive statistics in tables and graphs. To determine differences in the association between different demographic variables, we performed Pearson’s Chi-Square analysis or Fisher’s exact test depending on which was appropriate. A multivariate logistic regression model was used to determine independent predictors of self-medication. Crude and adjusted Odd Ratios (ORs) together with their 95% confidence intervals are reported. All variables from the univariate logistic regression were added to the model regardless of their significance. A p-value of 0.05 was considered statistically significant.

### 2.6 Ethical consideration

Ethical clearance was obtained from the Tropical Diseases Research Centre (TDRC) ethics committee (IRB No. 0000291) and National Health Research Authority (NHRA). The study was of minimal harm to participants as it was non-interventional. All data were anonymised and no identifiers were collected. Access to study data was restricted to investigators to ensure confidentiality.

## 3 Results

### 3.1 Prevalence of self-medication by demographic characteristics of participants

A total of 334 students participated in our study. There were slightly more female students (n = 168). The majority of the students were between 18 and 24 years of age (n = 200), single (n = 315), and/or belonged to the Christian religion (n = 330). There was an overall difference in self-medication practices with respect to the year of study (p = 0.038).

### 3.2 Reasons for self-medication

There was a higher proportion of students who reported self-medicating because of long queues at the hospital (n = 251, 75%) and not finding time to visit the hospital when not feeling well (n = 218, 65%). A lower proportion of students reported the rudeness of resident nurses and doctors to medical students as a reason for self-medicating (n = 76, 22.8%).

### 3.3 Class of medicines used in self-medication

**Fig. 1:**
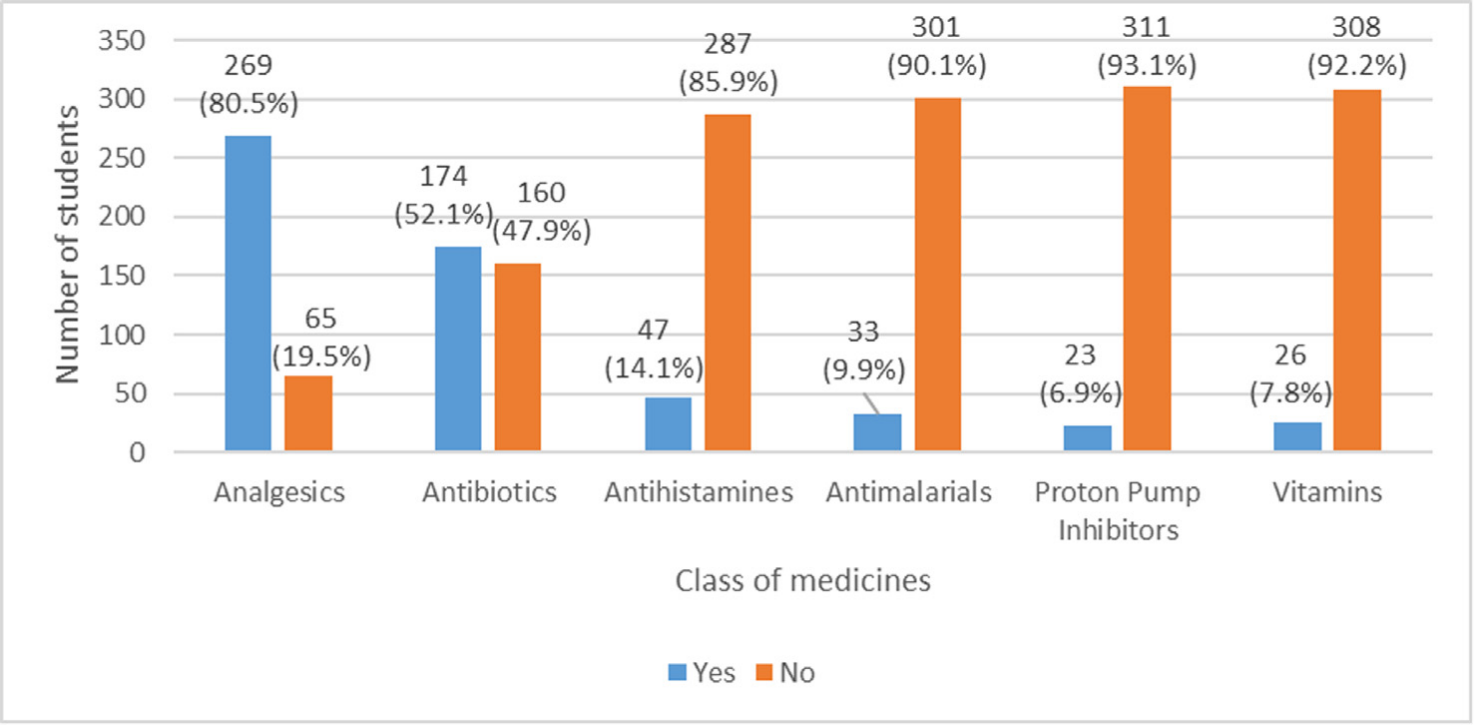
Class of medicines used in self-medication.

The most commonly used drug classes were analgesics (n = 269, 80.5%) followed by antibiotics (n = 174, 52.1%). The least used drug class were proton pump inhibitors (n = 23, 6.9%) (Fig. 1).

### 3.4 Independent predictors of self-medication

In bivariate analysis, only being in the fourth year of study increased the likelihood of self-medicating [OR:2.76; 95% CI (1.33–5.74)]. Other variables were not significant. Similarly, when adjusted for other factors, a student in the fourth year of study was>3 times likely to self-medicate [AOR:3.43; 95% CI (1.52–7.73)] (Table 3).

**Table 3:** Bivariate and multivariate logistic regression of factors associated with self-medication.

**Variable**	**Total**	**Self-Medication**	**Crude OR (95% CI)**	**Adjusted OR (95% CI)**
	n	yes (n, %)		
**Age**				
18–24 years	200	122 (61.0)	1	1
>24 years	134	82 (61.2)	0.99 (0.63–1.55)	0.73 (0.40–1.34
**Gender**				
Male	166	105 (63.2)	1	1
Female	168	99 (58.9)	1.20 (0.772–1.86)	1.15 (0.72–1.85)
**Marital Status**				
Single	315	192 (61.0)	1	1
Married	17	10 (58.8)	a	a
Divorced	2	2 (100)	a	a
**Religion**				
Christian	330	202 (61.2)	1	1
Muslim	4	2 (50.0)	1.58 (0.22–11.3	1.45 (0.20–10.76)
**Year of Study**				
2nd Year	59	33 (55.9)	1	1
3rd Year	55	43 (78.2)	0.98 (0.52–1.83)	1.24 (0.59–2.62)
4th Year	43	29 (67.4)	2.76 (1.33–5.74)	3.43 (1.52–7.73)
5th Year	53	29 (54.7)	0.60 (0.77–3.32)	1.91 (0.85–4.25)
6th Year	124	70 (56.5)	0.93 (0.49–1.78)	1.01 (0.52–1.98)

^a^Multivariate estimates were left out because they were too small to be interpreted.

## 4 Discussion

We assessed self-medication among medical students from Copperbelt University including the reasons for self-medication and the most self-prescribed drugs. Our study demonstrated that 61% of the students self-medicate, similar to studies conducted in Mansoura in Egypt (62.9%) and Ethiopia (59.7%) (Helal and Abou-ElWafa, 2017, Tesfaye et al., 2020).

In this study sex, age, marital status, and religion were not significantly associated with self-medication among medical students at the university. However, self-medication was significantly associated with the year of study (p < 0.038). Students in the 3rd and 4th year reported having self-medicated the most compared to students in the 2nd, 5th, and 6th year. These findings were different from other studies done in Southwestern Nigeria and the Netherlands which found that self-medication increased from the first level of study to the final year (Osemene and Lamikanra, 2012, Pandya et al., 2013, Van der Veer et al., 2011).

The reasons for self-medication reported were mainly due to long queues at the hospitals (75.1%) and lack of time to visit the hospital (65.3%). This is probably due to the fact that students require a lot of time to attend lectures and study. The rudeness of the resident doctors and nurses was found to be a less likely reason for self-medication. Other uncommon reasons included medical students having enough knowledge about health problems and therefore treating the health conditions themselves, shortage of medicines in hospitals, and long distances to the hospital.

The drug classes that are were often used by the students in this study were analgesics (80.5%) followed by antibiotics (52.1%) and proton pump inhibitors (6.9%) were reported to have the lowest prevalence. Studies by Pirzadeh and Mostafavi (Pirzadeh and Mostafavi, 2014), Zewdie (Zewdie et al., 2020), and Tesfaye (Tesfaye et al., 2020) also found that analgesics were mostly self-administered drugs. In contrast, the use of analgesics was higher in our study compared to these other studies. The students in our study reported using analgesics to treat headaches and other symptoms associated with pain. Antibiotics were the second most common group of drugs used to self-medicate. Antimicrobial resistance, a global health problem, is a huge risk in the self-medication of antibiotics (Rather et al., 2017). The self-medication with antibiotics is also compounded by the fact that antibiotics are easily accessible in many LMICs and often sold without a prescription (Sulis and Gandra, 2021).

The predictors of self-medication that were investigated included age, gender, religion, marital status, and the year of study. Being in the fourth year of study was the only predictor that increased the likelihood of self-medicating by 3 times. This result may be attributed to the fact that students currently learn pharmacology and therapeutics in the 2nd and 3rd year and therefore feel sufficiently knowledgeable about medicines by the 4th year which also happens to be the start of a clinical year. As students get to the 5th and 6th years, they would have learned about rational drug use in their clinical years hence the slight reduction in self-medicating behaviours. Unlike the study by Klemenc-Ketis (Klemenc-Ketis et al., 2010) and Araia (Araia et al., 2019), our study found no significant difference in self-medication between males and females, a finding similar to another study by Tesfaye (Tesfaye et al., 2020).

## 5 Conclusions

In conclusion, self-medication was found to be prevalent among medical students from the Copperbelt University. Analgesics and antibiotics were the most common classes of drugs that were used to self-medicate. In this study, the year of study was found to be an independent predictor for self-medication practice. Students should therefore be educated on the consequences of self-medication practices especially the irrational use of antibiotics. Further studies should also be conducted to assess the practice in other students other than medical students as well as in the general population.

## Funding

This research did not receive any specific grant from funding agencies in the public, commercial, or not-for-profit sectors.

**Author contributions**

Conceptualisation: OB, SKM; data and statistical analysis: OB, VD; Writing: OB, SKM, PVM, VD; Review of the manuscript: SKM, PVM, VD.

**Role of the funding source**

The authors declare that no funding was received.

**Data sharing statement**

The data that support the findings of this study are available from the corresponding author upon reasonable request.

### CRediT authorship contribution statement

**Owen Banda:** Conceptualization, Methodology, Investigation, Formal analysis, Writing - original draft. **Pipina Anna Vlahakis:** Formal analysis, Writing - Review and Editing. **Victor Daka:** Methodology, Formal analysis, Writing - Review and Editing. **Scott Kaba Matafwali:** Conceptualization, Methodology, Investigation, Writing - Review and Editing, Supervision.

## Declaration of Competing Interest

The authors declare that they have no known competing financial interests or personal relationships that could have appeared to influence the work reported in this paper.
